# Phytocannabinoids Profile in Medicinal Cannabis Oils: The Impact of Plant Varieties and Preparation Methods

**DOI:** 10.3389/fphar.2020.570616

**Published:** 2020-11-13

**Authors:** Michele Dei Cas, Eleonora Casagni, Antonella Casiraghi, Paola Minghetti, Diego Maria Michele Fornasari, Francesca Ferri, Sebastiano Arnoldi, Veniero Gambaro, Gabriella Roda

**Affiliations:** ^1^ Department of Health Sciences, Università Degli Studi di Milano, Milan, Italy; ^2^ Department of Pharmaceutical Sciences, Università Degli Studi di Milano, Milan, Italy; ^3^ Department of Medical Biotechnology and Traslational Medicine, Universitá degli Studi di Milano, Milan, Italy

**Keywords:** cannabinoids, medical cannabis, chemometrics methods, pharmaceutical chemistry, phytochemistry

## Abstract

Cannabis (*Cannabis sativa* L.) is a highly promising medicinal plant with well-documented effectiveness and growing use in the treatment of various medical conditions. Cannabis oils are mostly used in galenic preparations, due to their easy adjustment of the administration dose, together with the enhanced bioavailability of its active compounds. As stated by the Italian Law (9/11/2015, 279 Official Gazette), “to ensure the quality of the oil-based cannabis preparation, the titration of the active substance(s) should be carried out.” This study aims to represent the Italian panorama of cannabis oils, which were analyzed (8,201) to determine their cannabinoids content from 2017 to 2019. After application of the exclusion criteria, 4,774 standardized cannabis oils were included, which belong to different medicinal cannabis varieties and prepared according to different extraction methods. The concentration of the principal cannabinoids was taken into account dividing samples on the basis of the main extraction procedures and cannabis varieties. According to this analysis, the most substantial variations should be attributed to different cannabis varieties rather than to their extraction protocols. This study may be the starting point of preparatory pharmacists to assess the correct implementation of the preparation procedures and the quality of the extracts.

## Introduction

The therapeutic benefits of cannabis are more and more recognized at the scientific level (Bar-Lev Schleider et al., 2018; Freeman et al., 2019; Levinsohn and Hill, 2020), and regulation have to consider the evolution of its use (Zaami et al., 2018; Corli et al., 2019; Brunetti et al., 2020). There are several listed medical indications in Italy, which should be treated accordingly with different cannabis varieties containing tetrahydrocannabinol (THC), cannabidiol (CBD), or both of them (Law 9/11/2015, 279 Official Gazette; Ministero della Salute, 2017; EMCDA, 2018).

Cannabis with high THC levels (Bedrocan) is used to treat conditions, such as Tourette’s syndrome (Black et al., 2019), glaucoma (Novack, 2016; Panahi et al., 2017), and nausea (Schussel et al., 2018). Pain reduction and muscle spasm (Whiting et al., 2015) should be handled with a combination of THC and CBD, which occur in Bediol. CBD reduces pain, inflammation, and psychoactive side effects of THC (Boyaji et al., 2020). Bedrolite mainly contains CBD and is employed in the treatment of various forms of epilepsy (Rosenberg et al., 2015; Gaston and Friedman, 2017; Brodie and Ben-Menachem, 2018; Office of Medicinal Cannabis, 2019).

Cannabis oil is the preparation form receiving more attention recently (Pacifici et al., 2017; Carcieri et al., 2018; MacCallum and Russo, 2018; Pacifici et al., 2018; Bettiol et al., 2019; Deidda et al., 2019; Mudge and Brown, 2019; Pacifici et al., 2019; Pegoraro et al., 2019) due to its easy adjustment of the needed individual administration dose along the treatment period, together with the enhanced bioavailability of its active compounds.

As stated by the Italian Law (9/11/2015, 279 Official Gazette) “to ensure the quality of the oil-based cannabis preparation, the titration of the active substance(s) should be carried out with sensitive and specific methodologies, such as liquid or gas chromatography coupled with the mass spectrometry and the extraction method must be authorized in accordance with of the legislation in force (Law 9/11/2015, 279 Official Gazette).

In this framework, considering the activity of our laboratory in the field of drugs of abuse in particular cannabis derivatives, synthetic cannabinoids and cathinones (Valoti et al., 2012; Cannizzaro et al., 2016) we were interested in studying the Italian panorama of cannabis oils (n. 8201 samples from 2017 to 2019), which were analyzed by our laboratory to determine their cannabinoids content. These oil samples belonging to different cannabis varieties, here intended as chemotypes (Dei Cas et al., 2020), principally contain THC (chemotype I: Bedrocan) or CBD (chemotype III: Bedrolite) or both of them (chemotype II: FM2 and Bediol). Italian pharmacists prepared them according to different extraction methods present in the scientific literature [Romano and Hazekamp, 2013; Citti et al., 2016; Società Italiana Farmacisti Preparatori (SIFAP), 2016; Calvi et al., 2018; Casiraghi et al., 2018]. The crucial step in the preparation method is the decarboxylation to transform THCA and CBDA, present in the plant material, in the corresponding neutral forms THC and CBD. The need for optimizing and standardizing decarboxylation procedures is dictated by pharmacological reasons because the acidic and neutral cannabinoids have different pharmacodynamic and pharmacokinetic properties that will influence the pharmacological profile of the final product, according to the relative amount of the two compounds. A striking pharmacokinetic difference between THCA and THC concerns the passage through the blood–brain barrier (BBB). As THCA is a substrate of P-glycoprotein (P-gp/abcb1) and breast cancer resistance protein (Bcrp/abcg2), its penetration into the CNS is limited (Spiro et al., 2012). Both abcb1 and abcg2 belong to the ATP-binding cassette (ABC) family of efflux transporters and are critical to BBB function, where they impede the passage of their substrates into the brain (Agarwal and Elmquist, 2012). Thus, the pharmacological activity of THCA would mainly rely on peripheral effects, as already suggested by the lack of psychoactive properties. This is not in contrast with the supposed antiemetic properties of THCA because some peripheral mechanisms of cannabinoids have been described. However, other proposed pharmacological effects of THCA, strictly related to central activities, such as muscle relaxation, should be reconsidered or refused (Russo, 2018).

The authors would like to highlight possible relationships among cannabis varieties, the effects of the extraction method, and the cannabinoids profile to better understand pharmacological activity of cannabis oils in clinical trials, as a function of oil composition, because very little information in the literature is reported about them. Moreover, it could be helpful for pharmacists, involved in the preparation of these medicines, to check the quality of their preparations. In fact, due to a lack of a single and standard preparation procedure, pharmacists very often ask for preprocessed cannabinoids concentrations to deal with.

## Materials and Methods

### Chemicals and Reagents

Methanol (MeOH), toluene, O, N-bis (trimethylsilyl) trifluoroacetamidetrimethylchlorosiloxane (BSTFA-1% TMCS), methyl oleate (99% purity), THC 1 mg/ml in MeOH (purity ≥ 95.0%), CBD 1 mg/ml in MeOH (purity ≥ 95.0%), and CBN 1 mg/ml in MeOH (purity ≥ 95.0%) were purchased from Sigma-Aldrich. The acidic forms of cannabinoids, such as THCA 1 mg/ml in acetonitrile (purity ≥ 95.0%) and CBDA 1 mg/ml in acetonitrile (purity ≥ 95.0%), were obtained from Cayman Chemical Company.

### Galenic Preparations

Cannabis oil galenic preparations were delivered for cannabinoids determination to our laboratory between 2017 and 2019 and account for 8,201 samples. However, after the initial data collection and laboratory analysis, samples were excluded on the basis of 1) the absence, in the detailed sheet, of pharmaceutical-grade *Cannabis sativa* varieties; 2) the use of pharmaceutical-grade *Cannabis sativa* varieties diverse from Bedrocan, Bediol, Bedrolite, and FM2; and 3) a nonstandardized preparation method. Consequently, this study was limited to 4,774 samples standardized for both pharmaceutical-grade cannabis varieties and the extraction methods. Preparation methods are mainly based on maceration of vegetable materials in olive oil at high temperature, at about 100°C or more [Methods A (Romano and Hazekamp, 2013) and B (Citti et al., 2016)]. Both of them do not require a preliminary decarboxylation of the vegetal matrix. A preliminary decarboxylation step is performed with Method C [Società Italiana Farmacisti Preparatori (SIFAP), 2016; Casiraghi et al., 2018] or Method D (Calvi et al., 2018). All these methods were used by pharmacists, based on medical prescriptions, to obtained cannabis oils by different varieties of medicinal grade plant material: the Dutch Bedrocan, Bediol, Bedrolite, and the Italian FM2. After decarboxylation, where planned, the cannabis decoctions in oil were mainly carried out with a weight-to-volume ratio between plant material and oil of 1:10 (usually 5 g in 50 ml) (Baratta et al., 2019). Mainly, pharmacopeia grade olive oil, usually virgin or refined according to the European Pharmacopoeia (Ph. Eur.), was used as extraction solvent. This oil can minimize the formation of large amounts of aldehydes and ketones that can also influence the digestibility of the macerated oil (Pavlovic et al., 2018).

### Analytical Samples Preparation From Cannabis Oils

Cannabis oil preparation (50 mg weighted) was added to 5 ml of methanol. The mixture was extracted by vortex and centrifuged (1789 × *g*, 5 min). Then, 50 µl of the supernatant was withdrawn and added with 50 µl of the internal standard solution (methyl oleate, 175 μg/ml in MeOH). The solvent was evaporated, then 50 µl of BSTFA-1% TMCS and 50 µl of toluene were added. The mixture was mixed and heated at 70°C for 30 min, to allow the derivatization.

### Analysis of Cannabinoids by GC/MS

The analyses were performed on a 5973 Hewlett Packard GC system, with a split–splitless injection system and an MS detector (Hewlett Packard) operated in the electron ionization (EI) mode (70 eV), as already described elsewhere (Casiraghi et al., 2018). Briefly, the GC was equipped with a capillary column Rxi-5ms (30 m × 0.25 mm, i.d. 0.25 mm, Restek). The GC/MS conditions were as follows: helium was used as the carrier gas at a flow rate of 1.2 ml/min, splitless mode (0.25 min); injector temperature 280°C; interface transfer line 300°C; ion source 230°C; and oven temperature program: initial 70°C, 40°C/min up to 180°C, then 10°C/min up to 300°C (6.25 min). The total analysis time was 21 min. The MS detector was operated in selected ion monitoring (SIM) acquiring characteristic ions in prefixed temporal windows each corresponding to a peculiar cannabinoid: IS methyl oleate at 8.5 min (264 m/z); CBD-2TMS at 9.7 min (390 m/z); THC-1TMS at 10.7 min (386 m/z); CBN-1TMS at 11.4 min (367 m/z); CBDA-3TMS at 11.7 min (491 m/z); and THCA-2TMS at 12.9 min (487 m/z)*.* Throughout this article, the concentrations of phytocannabinoids were expressed as percentage weight per weight (% w/w, weight of cannabinoid/weight of oil preparation).

### Statistical Analysis

Descriptive statistics was investigated using GraphPad Prism 7.0 (GraphPad Software, Inc., La Jolla, CA). In order to find out potential discriminating features between the groups, a series of univariate and multivariate analyses were performed using the software MetaboAnalyst 4.0. The groups were designed considering cannabis varieties (Bedrocan, Bediol, FM2, and Bedrolite) and the extraction protocol [Methods A (Romano and Hazekamp, 2013), B (Citti et al., 2016), C (Società Italiana Farmacisti Preparatori (SIFAP), 2016; Casiraghi et al., 2018), and D (Calvi et al., 2018)]. Data were checked for integrity, filtered by interquartile range, log-transformed (generalized log transformation), and mean centered. PCA and hierarchical clustering with heatmap were used for considering all variables in the data set simultaneously. In the heatmap analysis, the clustering algorithm was set to Ward, and the distance measure to Euclidean. VIP scores, resulting from the supervised PLS-DA analysis, were used as a cutoff (>1) to include variables with discriminatory power. Further investigations were completed by ANOVA coupled to post hoc Fisher’s LSD test to highlight the significant variables with a threshold *p*-value of <0.05.

## Results

**FIGURE 1:**
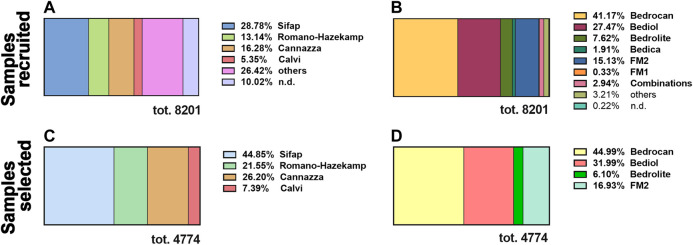
The distribution, between 2017 and 2019, of the total amount of cannabis oil extracts recruited by our laboratory (8,201) by preparation methods **(A)** and varieties of *Cannabis sativa* **(B)**. The distribution of standardized cannabis oil extracts selected for this study (4,774) by preparation methods **(C)** and varieties of *Cannabis sativa* **(D)**. n.d. not determined since those details were not indicated in the sample’s addendum. For details on preparation methods, see the following references: Method A (Romano and Hazekamp, 2013), Method B (Citti et al., 2016), Method C (Società Italiana Farmacisti Preparatori (SIFAP), 2016; Casiraghi et al., 2018), and Method D (Calvi et al., 2018).

From 2017 to 2019, 8,201 samples of cannabis olive oils were delivered to our laboratory for cannabinoid level determination. Samples were time-distributed as follows: in 2017, 1,349 (16.5%), in 2018, 2,281 (27.8%), and in 2019, 4,571 (55.7%). Cannabis oils were divided by preparation methods (Figure 1A) and varieties of *Cannabis sativa* (Figure 1B).

The most used maceration technique for the oil extraction of cannabinoids was Method C (28.8%), followed by Method B (16.3%) and Method A (13.1%). The more prevalent medical cannabis chemotypes comprised Bedrocan (41.2%), Bediol (27.4%), and the Italian FM2 (15.1%).

All the further statistical analysis were restricted only to a well-characterized subpopulation made of 4,774 (58% of the entire population of 8,201) excluding samples (42%, 3,457) that were not accompanied by a detailed sheet or are not standardized as regard cannabis varieties and method preparation. In the same way, the selected population was divided by preparation methods (Figure 1C) and varieties of *Cannabis sativa* (Figure 1D). The subpopulation sampled maintains the same distribution of the preparation methods and plant varieties with respect to the total.

**FIGURE 2:**
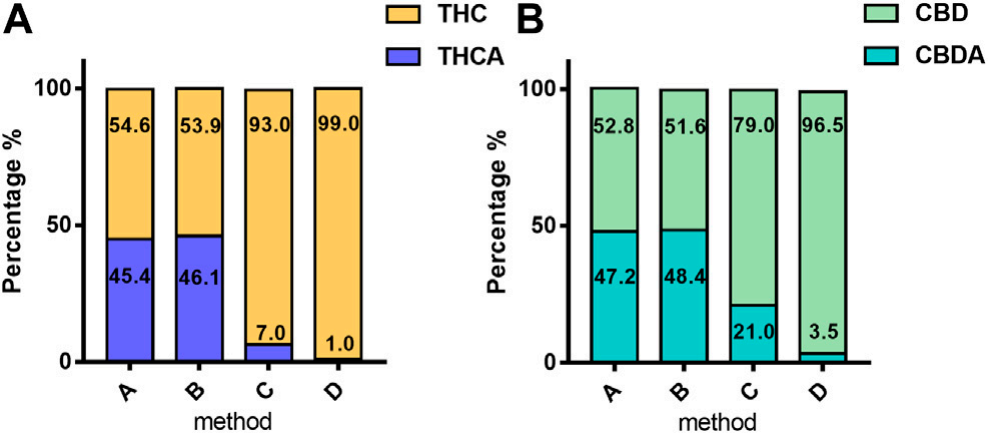
Mean percentage of acidic and neutral form of phytocannabinoids in 4,774 samples according to the extraction method: **(A)** THC and THCA; **(B)** CBD and CBDA. The values are expressed as mean normalized to 100: % acidic form = [Mean_acid_/(Mean_acid_ + Mean_neutral_)] × [100/(Mean_acid_ + Mean_neutral_)]; % neutral form = [Mean_neutral_/(Mean_acid_ + Mean_neutral_)] × [100/(Mean_acid_ + Mean_neutral_)]. For details on preparation methods, see the following references: Method A (Romano and Hazekamp, 2013), Method B (Citti et al., 2016), Method C (Società Italiana Farmacisti Preparatori (SIFAP), 2016; Casiraghi et al., 2018), and (Method D (Calvi et al., 2018).

The main differences in the cannabinoid profile are due to the decarboxylation step and especially to the heating time and temperature applied. These differences are directly related to the percentage of acidic forms (Figure 2) of cannabinoids.

These forms, at high temperatures, are subjected to decarboxylation to respective neutral forms. Methods A and B showed a higher content of the acidic forms compared with the neutral ones from 90 to 50% of the total content of cannabinoids (THC + THCA; CBD + CBDA). In particular, the extraction without a decarboxylation step (Method A: 98°C for 1 h and Method B 110°C for 2 h) leads to a highly variable ratio of acidic/neutral cannabinoids, thus reducing the reproducibility of the extraction procedure.

On the contrary, Methods C and D described a decarboxylation step (respectively, in the oven at 115°C for 40 min and 145°C for 30 min) before oil maceration with a full conversion of the acidic to neutral forms. Then, in Method C, the decarboxylated cannabis is extracted in oil heated by means of a water bath (100°C for 40 min), whereas in method D the extraction is carried out by ultrasound (35 kHz 30 min). In Method C, neutral forms of both THC and CBD were prevalently valued at 93% and 79%, respectively. Moreover, in Method D, the neutral forms covered almost the totality of the cannabinoids, THC 99%, and CBD 96.5%.

**FIGURE 3:**
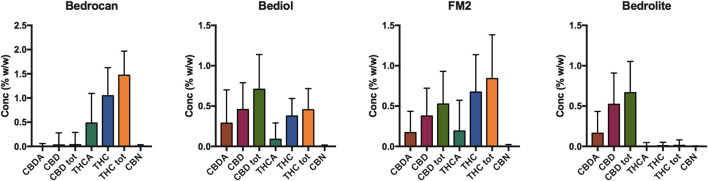
Distribution of phytocannabinoids among *Cannabis sativa* varieties (4,774, mean ± SD).

The distribution of phytocannabinoids among varieties (Figure 3) was further investigated. The detailed samples list separated by varieties and processing methods can be found in the Supplementary Tables S1–S4. Bedrocan displayed the highest content of total THC (mean ± SD, 1.47 ± 0.47), followed by FM2 (0.54 ± 0.12) and Bediol (0.45 ± 0.26), whereas Bedrolite, as expected, showed very low amounts of this cannabinoid (0.01 ± 0.09). The situation was the opposite when considering total CBD, in which the highest content was found in FM2 (0.89 ± 0.30), followed by Bediol (0.70 ± 0.45) and Bedrolite (0.66 ± 0.35). Bedrocan displayed, as expected, a slight concentration of CBD (0.04 ± 0.31).

In the different cannabis varieties, the total amount of THC and CBD (Supplementary Table S5) are similar to those declared in the literature (Uso medico della cannabis - Ministero della Sanità, 2016; Office of Medicinal Cannabis, 2019) and in labeled content. Some samples deviated from the expected values due to the variability in both the not strictly standardized preparation protocols and the employed plant matrix.

**FIGURE 4:**
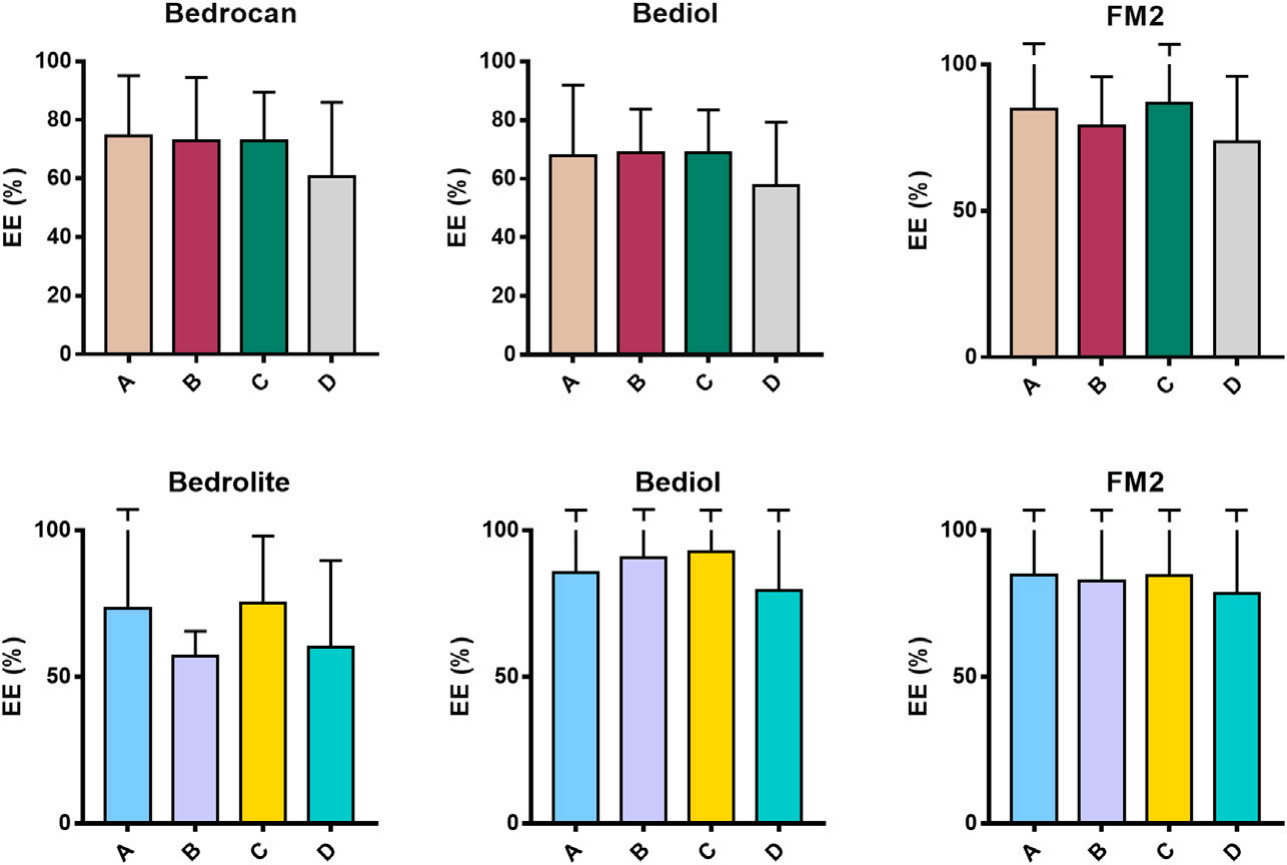
Extraction efficiency (EE%) of THC (**up**) and CBD (**down**) measured in cannabis oil samples (4,774) obtained using different cannabis varieties and preparation methods. The error bars that exceed the axis limit are represented as clipped. The theoretical extraction rate was set as the mean of the declared range content as follows: Bedrocan THC 2.05 (% w/w); Bediol THC 0.65 (% w/w), CBD 0.75 (% w/w); FM2 THC 0.65 (% w/w); CBD 1.05 (% w/w); and Bedrolite CBD 0.85 (% w/w). For details on preparation methods, see the following references: Method A (Romano and Hazekamp, 2013), Method B (Citti et al., 2016), Method C [Società Italiana Farmacisti Preparatori (SIFAP), 2016; Casiraghi et al., 2018)], and Method D (Calvi et al., 2018). The values are expressed as mean ± SD and calculated according to the equation EE% = (conc. Exp/conc. Theo) × 100.

**FIGURE 5:**
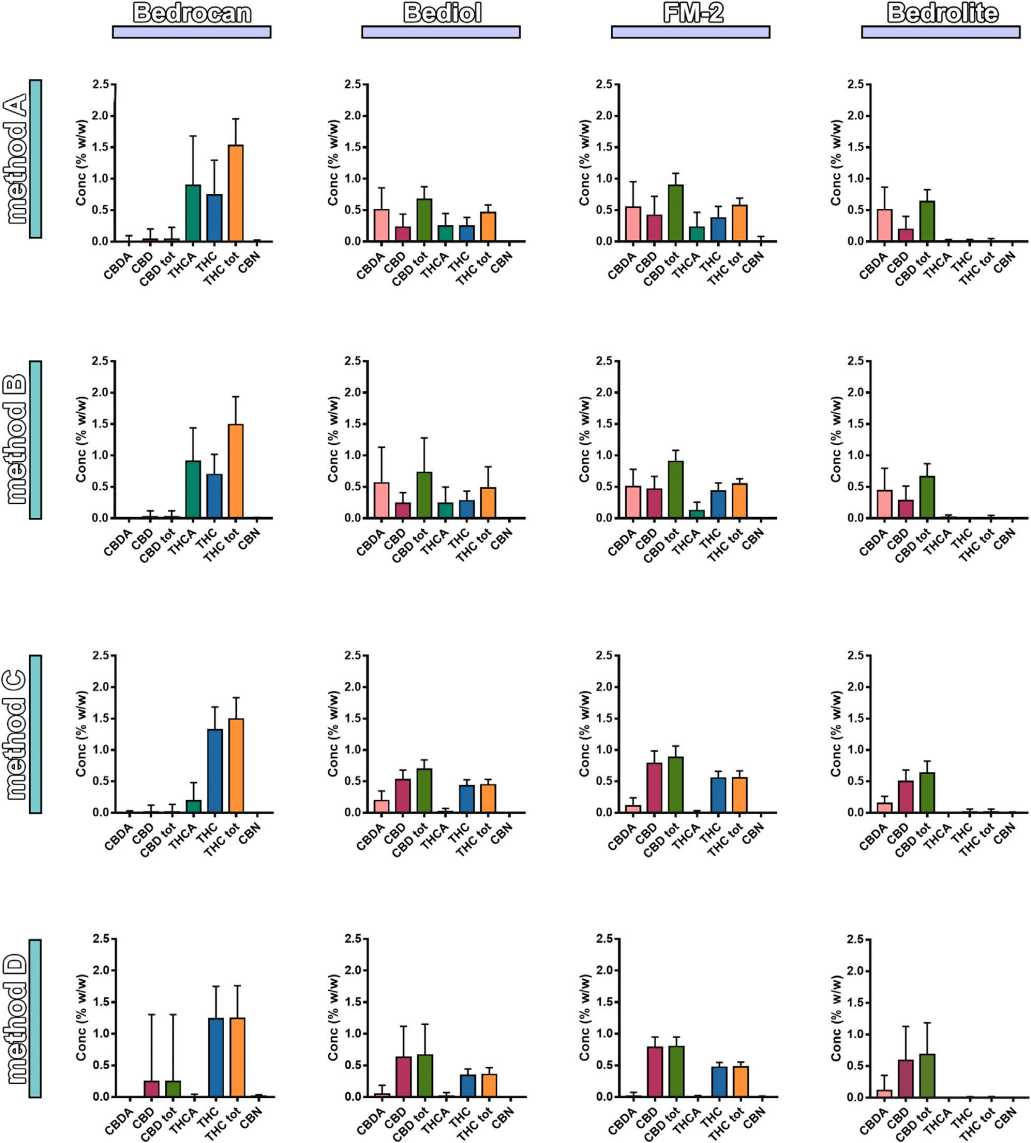
Distribution of phytocannabinoids among extraction methods from plant materials and varieties (4,774, mean ± SD). The columns represented the cannabis sativa varieties (sx to dx) Bedrocan, Bediol, FM2, and Bedrolite and the rows the Method of extraction (up to down) [Method A (Romano and Hazekamp, 2013), Method B (Citti et al., 2016), Method C (Società Italiana Farmacisti Preparatori (SIFAP), 2016; Casiraghi et al., 2018), and Method D (Calvi et al., 2018)].

Samples were also analyzed taking into consideration the efficiency of extraction of total THC and CBD depending on varieties and the preparation method (Figure 4 and Supplementary Table S6). Among all samples analyzed, a reduced number of results showed coherence among the preparation method and declared content of cannabinoids. As result, the extraction efficiency (EE%) ranges (min–max) were from 57.6 to 86.3 for THC and from 57.1 to 92.8% for CBD. Figure 5 and Table 1 illustrate the concentration of cannabinoids within main cannabis oil varieties (columns) processed with the most common methods (rows). Being confirmed that the total extracted content of THC and CBD is not significantly different with respect to the extraction method, it is interesting to note that, on the contrary, the relative content of the acidic or neutral form is strictly related to preparation method condition. Samples prepared according to Methods C and D showed a high level of neutral active THC form, whereas methods A and B results were in favor of THCA. The relative content of the two forms is essential for the expected pharmacological effect.

**TABLE 1:** Cannabinoids concentrations, expressed as both mean ± SD and 25–75th percentile range, as a function of preparation methods and varieties.

		THC tot (% w/w)		CBD tot (% w/w)	
Cannabis products	n	Mean ± SD	Range (25–75th)	Mean ± SD	Range (25–75th)
Bedrocan	2,148	1.47 ± 0.466	1.30–1.68	0.41 ± 0.313	—
Method A	515	1.53 ± 0.425	1.34–1.74	0.04 ± 0.185	—
Method B	682	1.49 ± 0.445	1.33–1.68	0.02 ± 0.096	—
Method C	800	1.49 ± 0.340	1.32–1.66	0.01 ± 0.119	—
Method D	151	1.24 ± 0.519	1.15–1.44	0.07 ± 0.544	—
Bedrolite	291	0.01 ± 0.091	—	0.66 ± 0.351	0.49–0.71
Method A	62	0.01 ± 0.036	—	0.64 ± 0.189	0.55–0.70
Method B	25	0.01 ± 0.034	—	0.66 ± 0.202	0.59–0.73
Method C	151	0.01 ± 0.045	—	0.63 ± 0.191	0.54–0.70
Method D	53	0.01 ± 0.011	—	0.68 ± 0.502	0.41–0.68
Bediol	1,527	0.45 ± 0.262	0.40–0.50	0.70 ± 0.445	0.60–0.76
Method A	253	0.46 ± 0.122	0.40–0.51	0.67 ± 0.203	0.58–0.75
Method B	350	0.48 ± 0.338	0.42–0.50	0.73 ± 0.552	0.64–0.74
Method C	838	0.44 ± 0.087	0.41–0.49	0.69 ± 0.149	0.62–0.79
Method D	86	0.35 ± 0.112	0.29–0.40	0.67 ± 0.486	0.46–0.64
FM-2	808	0.54 ± 0.120	0.47–0.63	0.89 ± 0.294	0.76–1.01
Method A	199	0.57 ± 0.118	0.50–0.65	0.89 ± 0.192	0.78–1.03
Method B	194	0.54 ± 0.085	0.51–0.60	0.91 ± 0.176	0.79–1.00
Method C	352	0.56 ± 0.111	0.49–0.63	0.88 ± 0.183	0.75–1.02
Method D	63	0.47 ± 0.077	0.42–0.52	0.80 ± 0.151	0.72–0.89

For details on preparation methods see the following references: Romano-Hazekamp [Method A (Romano and Hazekamp, 2013)], Cannazza [Method B (Citti et al., 2016)], Sifap [Method C (Società Italiana Farmacisti Preparatori (SIFAP), 2016; Casiraghi et al., 2018)], and Calvi [Method D (Calvi et al., 2018)].

**FIGURE 6:**
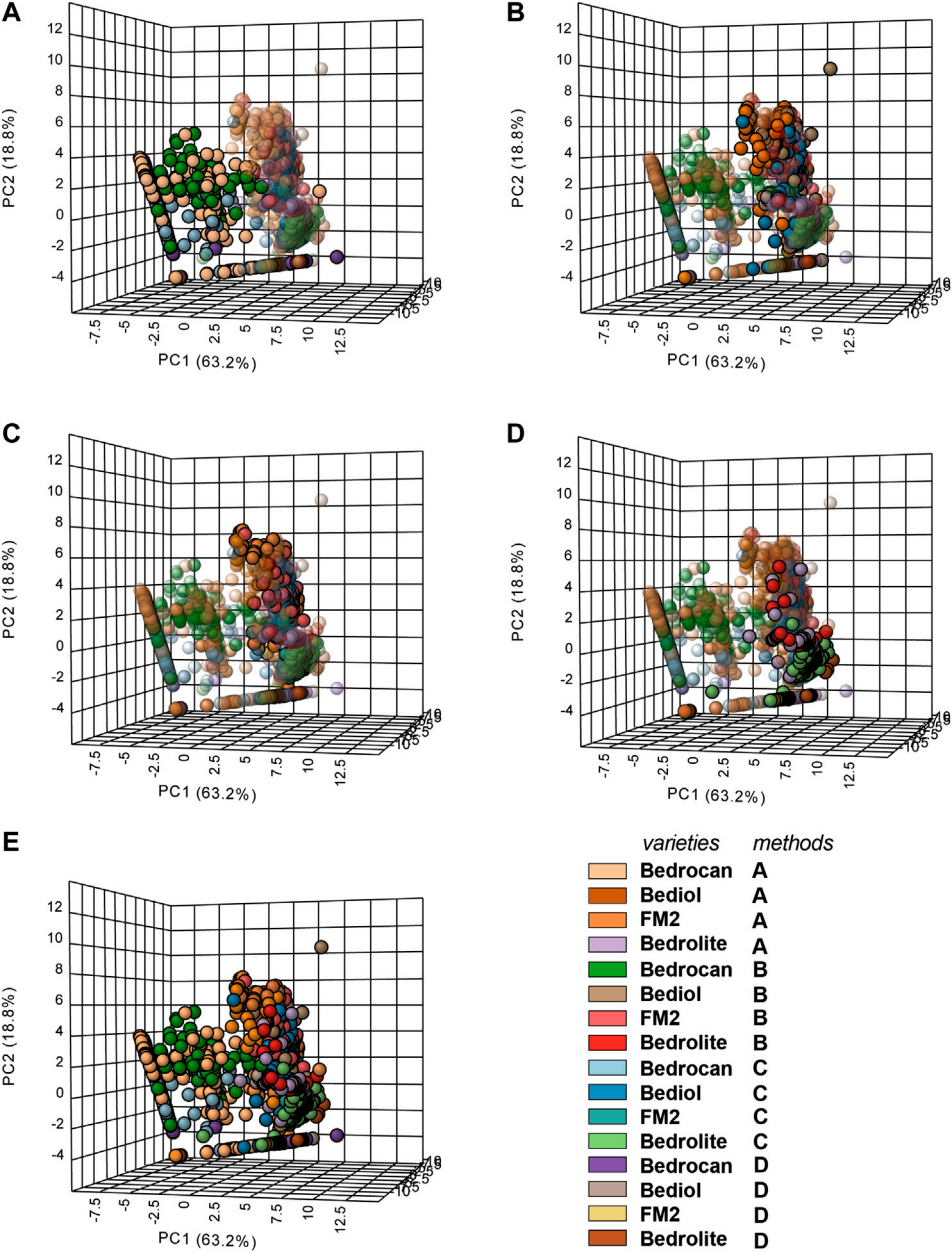
3D Principal component analysis (PCA) plot of cannabis oil extracts divided into groups according to the plant varieties and extraction method (4,774). In the panel, the plant varieties are evidenced, whereas the extraction adopted was color coded (according to the legend). In the panel, **(A)** Bedrocan, **(B)** Bediol, **(C)** FM2, **(D)** Bedrolite, and **(E)** the entire data set overview are evidenced. For details on preparation methods, see the following references: Method A (Romano and Hazekamp, 2013), Method B (Citti et al., 2016), Method C [Società Italiana Farmacisti Preparatori (SIFAP), 2016; Casiraghi et al., 2018], and Method D (Calvi et al., 2018).

Multivariate analysis (Figures 6 and Supplementary Figure S1) showed only an appreciable separation between Bedrocan and other varieties, Bediol, Bedrolite, and FM2, which were not well detached among them.

**FIGURE 7:**
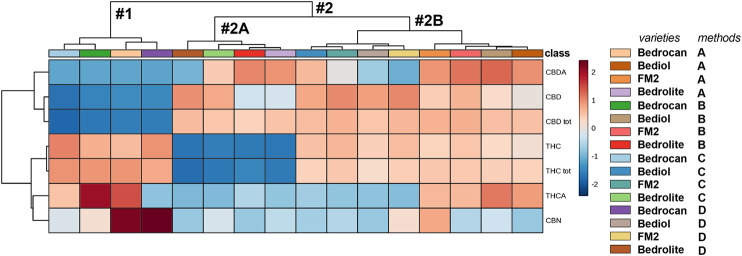
A heatmap overview (showing only group average) with hierarchical clustering of the 4,774 cannabis oils. The first cluster (#1) included Bedrocan variety and the second one (#2) the other varieties, which in particular consisted of (#2A) Bedrolite and (#2B) Bediol and FM2. In respect to other varieties, Bedrocan displayed a lower concentration of CBD (tot, neutral, and acid) and Bedrolite of THC (tot and neutral). The color-scale differentiates values as high (red), mid (gray), and low (blue). For details on preparation methods, see the following references: Method A (Romano and Hazekamp, 2013), Method B (Citti et al., 2016), Method C [Società Italiana Farmacisti Preparatori (SIFAP), 2016; Casiraghi et al., 2018], and Method D (Calvi et al., 2018).

The same conclusion can be found in Figure 7, which shows a heatmap coupled to hierarchical clustering, in which the cannabinoids profile is graphed against plant varieties and extraction protocol. The map is color coded to three concentration levels (blue = low, gray = middle, and red = high range). Hierarchical clustering is a frequently used method to identify similarities or differences between each individual. We noted the presence of two different and well-divided clusters, represented as dendrogram: one including Bedrocan variety and the second one included other varieties. The latter consisted of two other clusters: Bedrolite and Bediol + FM2. In respect to other varieties, Bedrocan displayed a lower concentration of CBD (tot, neutral, and acid) along with a higher concentration of THCA and CBN, whereas Bedrolite presented a weaker concentration of THC (total and neutral). As clearly demonstrated (Figures 6, 7 and Supplementary Figure S1), the formation of subgroups within the data set can only be done based on the variety of cannabis inflorescence and not by the extraction methods. PCA is not always able to properly separate the variations produced by each factor, and the results can be somehow problematic to read. In order to avoid this scenario, univariate and supervised statistical tests were also performed. The use of a more conservative method (ANOVA, *post hoc* Fisher’s LSD) demonstrated that all the considered cannabinoids should be capable (*p* < 0.05) of discriminating against groups. THC, which showed a VIP score of 1.71 and a *p* value <0.05, was therefore proposed as the best phytocannabinoid able to discriminate between cannabis oils extracted by different methods and coming from different varieties (Supplementary Figure S2). However, as mentioned above, the most substantial variations should be attributed to the different cannabis varieties rather than to their extraction protocols. Further considering the extraction method results, different amplitudes of variability can be observed: higher values were reported in Methods A and B with respect to Methods C and D. The more strictly standardized preparation protocols of the latest are therefore useful.

## Discussion

Medical cannabis has been effectively used for treating symptoms from a variety of disorders. Commonly, it is prescribed when first-choice treatments and medicines are not effective enough or have severe side effects. Despite the growing popularity of cannabis-based medicinal oils (Pacifici et al., 2017; Carcieri et al., 2018; Pacifici et al., 2018; Bettiol et al., 2019; Deidda et al., 2019; Mudge and Brown, 2019; Pacifici et al., 2019; Pegoraro et al., 2019), at the moment, there are no studies in which the cannabinoid composition has been strictly defined considering the variety of the plant and the extraction method. However, a notable contribution in this research field comes from the National Institute of Health in Italy, who was involved in the determination of long-term stability of cannabinoids in standardized cannabis oils to assure their quality and therapeutic properties (Pacifici et al., 2017; Pacifici et al., 2018; Pacifici et al., 2019). The relevance of these studies lies in ensuring a conscious prescription by the physicians, who should take into consideration both the composition and stability of cannabis oils. Nevertheless, from a pharmacological point of view, the composition of the final product in THCA and THC content is critical, being the THCA activity mainly based on peripheral effects and, therefore, much less impressive in the majority of situations. Our results stated that cannabinoid content are significantly linked to cannabis varieties (i.e., Bedrocan, Bedrolite, Bediol, and FM2), among which pharmacists and physicians can choose the most suitable. Moreover, there is a clear trend in cannabinoid content with respect to the preparation methods. It is interesting to note that total THC and CBD extracted amounts were in the same range, whereas those methods with the preliminary decarboxylation step (Method C and D) allowed obtaining oils richer in the active neutral form. For these reasons, this study may be the starting point for compounded oils in pharmacies to assess the correct implementation of the preparation procedures and the quality of the extracts. However, there are still many aspects to be improved, including the standardization of raw inflorescences and oil extraction procedures.

## Data Availability Statement

The original contributions presented in the study are included in the article/Supplementary Material, further inquiries can be directed to the corresponding author/s.

## Author Contributions

Conceptualization: MDC and GR. Investigation: FF, SA, and EC. Formal analysis and drafting of the manuscript: MDC. Supervision: GR, VG, and PM. Writing—review and editing: EC, AC, PM, DF, and GR. All authors have read and agreed to the published version of the manuscript.

## Funding

This research did not receive any specific grant from funding agencies in the public, commercial, or not-for-profit sectors.

## Conflict of Interest

The authors declare that the research was conducted in the absence of any commercial or financial relationships that could be construed as a potential conflict of interest.
