# Supplementary material for: Phytocannabinoids Profile in Medicinal Cannabis Oils: The Impact of Plant Varieties and Preparation Methods
Source: Front Pharmacol. 2020 Nov 13;11:570616. doi: 10.3389/fphar.2020.570616 (PMC7751640; doi:10.3389/fphar.2020.570616)
Supplement: Supplementary file 1 [file datasheet1.docx]

**Supplementary materials.
Phytocannabinoids profile in medicinal cannabis oils: the impact of plant varieties and preparation methods**

Michele Dei Cas^1^, Eleonora Casagni^2^, Antonella Casiraghi^2^, Paola Minghetti^2^, Diego Fornasari^3^, Francesca Ferri^2^, Sebastiano Arnoldi^2^, Veniero Gambaro^2^, Gabriella Roda^2,*^

^1^ Department of Health Sciences, Università degli Studi di Milano, Via A. di Rudinì 8, 20142, Milan, Italy

^2^ Department of Pharmaceutical Sciences, Università degli Studi di Milano, Via L. Mangiagalli 25, 20133, Milan, Italy

^3^ Department of Medical Biotechnology and Translational Medicine, Via Vanvitelli 32, 20133, Milan, Italy

**Corresponding author**: Gabriella Roda. Department of Pharmaceutical Sciences, Università degli Studi di Milano, Via L. Mangiagalli 25, 20133, Milan, Italy. email: [gabriella.roda@unimi.it](mailto:gabriella.roda@unimi.it)

**Please be aware that the references in the supplementary materials are referred to those in the main text. Table S1.** Phytocannabinoids concentrations (% w/w, mean ±SD) in Cannabis sativa oil preparations obtained using Method A [26] for the extraction of analytes from plant materials. In the first column are presented the different Cannabis varieties or some combinations among them.

| **ROMANO HAZEKAMP (n.1061)** | **n.** | **CBDA** | **±SD** | **CBD** | **±SD** | **CBD tot** | **±SD** | **THCA** | **±SD** | **THC** | **±SD** | **THC tot** | **±SD** | **CBN** | **±SD** |
| --- | --- | --- | --- | --- | --- | --- | --- | --- | --- | --- | --- | --- | --- | --- | --- |
| BEDICA | 10 | 0.00 | 0.00 | 0.02 | 0.03 | 0.02 | 0.03 | 0.76 | 0.50 | 0.49 | 0.43 | 1.16 | 0.11 | 0.00 | 0.00 |
| BEDIOL | 253 | 0.50 | 0.35 | 0.23 | 0.21 | 0.67 | 0.20 | 0.25 | 0.20 | 0.25 | 0.14 | 0.46 | 0.12 | 0.00 | 0.00 |
| BEDROCAN | 515 | 0.01 | 0.09 | 0.04 | 0.16 | 0.04 | 0.18 | 0.89 | 0.79 | 0.75 | 0.56 | 1.53 | 0.42 | 0.01 | 0.02 |
| BEDROCAN + BEDROLITE | 5 | 0.51 | 0.06 | 0.00 | 0.01 | 0.45 | 0.06 | 0.44 | 0.11 | 0.03 | 0.04 | 0.42 | 0.09 | 0.01 | 0.03 |
| BEDROCAN + CBD | 1 | 0.00 | 0.00 | 0.58 | 0.00 | 0.58 | 0.00 | 1.01 | 0.00 | 0.55 | 0.00 | 1.43 | 0.00 | 0.00 | 0.00 |
| BEDROLITE | 62 | 0.51 | 0.36 | 0.20 | 0.21 | 0.64 | 0.19 | 0.01 | 0.03 | 0.01 | 0.03 | 0.01 | 0.04 | 0.00 | 0.00 |
| FM1 | 1 | 0.00 | 0.00 | 0.00 | 0.00 | 0.00 | 0.00 | 0.84 | 0.00 | 0.28 | 0.00 | 1.01 | 0.00 | 0.00 | 0.00 |
| FM2 | 199 | 0.54 | 0.41 | 0.42 | 0.30 | 0.89 | 0.19 | 0.22 | 0.24 | 0.38 | 0.19 | 0.57 | 0.12 | 0.01 | 0.07 |
| PEDANIOS | 5 | 0.08 | 0.17 | 0.20 | 0.40 | 0.27 | 0.55 | 0.45 | 0.98 | 0.99 | 0.74 | 1.38 | 0.85 | 0.00 | 0.00 |
| High THC | 9 | 0.00 | 0.00 | 0.02 | 0.03 | 0.02 | 0.03 | 0.01 | 0.03 | 1.49 | 0.13 | 1.50 | 0.13 | 0.01 | 0.01 |

**Table S2.** Phytocannabinoids concentrations (% w/w, mean, and SD) in Cannabis sativa oil preparations obtained using Method B [27] for the extraction of analytes from plant materials. In the first column are presented the different Cannabis varieties or some combinations among them.

| **CANNAZZA (n. 1286)** | **n.** | **CBDA** | **±SD** | **CBD** | **±SD** | **CBD tot** | **±SD** | **THCA** | **±SD** | **THC** | **±SD** | **THC tot** | **±SD** | **CBN** | **±SD** |
| --- | --- | --- | --- | --- | --- | --- | --- | --- | --- | --- | --- | --- | --- | --- | --- |
| BEDICA | 8 | 0.00 | 0.01 | 0.00 | 0.00 | 0.00 | 0.01 | 0.36 | 0.43 | 0.74 | 0.27 | 1.05 | 0.25 | 0.00 | 0.00 |
| BEDIOL | 350 | 0.57 | 0.57 | 0.23 | 0.16 | 0.73 | 0.56 | 0.24 | 0.26 | 0.27 | 0.16 | 0.48 | 0.34 | 0.00 | 0.01 |
| BEDROBINOL | 7 | 0.00 | 0.00 | 0.00 | 0.00 | 0.00 | 0.00 | 1.47 | 0.71 | 0.31 | 0.16 | 1.60 | 0.77 | 0.00 | 0.00 |
| BEDROCAN | 682 | 0.00 | 0.01 | 0.02 | 0.10 | 0.02 | 0.10 | 0.91 | 0.53 | 0.69 | 0.32 | 1.49 | 0.44 | 0.00 | 0.01 |
| BEDROCAN + BEDROLITE | 6 | 0.42 | 0.37 | 0.03 | 0.04 | 0.40 | 0.35 | 0.89 | 0.13 | 0.32 | 0.28 | 1.10 | 0.36 | 0.00 | 0.00 |
| BEDROCAN + CBD | 5 | 0.01 | 0.01 | 0.67 | 0.25 | 0.67 | 0.25 | 0.75 | 0.15 | 0.53 | 0.31 | 1.19 | 0.41 | 0.00 | 0.00 |
| BEDROLITE | 25 | 0.54 | 0.35 | 0.17 | 0.18 | 0.66 | 0.20 | 0.06 | 0.20 | 0.02 | 0.10 | 0.01 | 0.03 | 0.00 | 0.00 |
| FM2 | 194 | 0.50 | 0.28 | 0.46 | 0.20 | 0.91 | 0.18 | 0.12 | 0.13 | 0.43 | 0.13 | 0.54 | 0.09 | 0.00 | 0.00 |
| FM2 + BEDIOL | 2 | 0.53 | 0.00 | 0.54 | 0.00 | 1.00 | 0.00 | 0.10 | 0.00 | 0.55 | 0.00 | 0.64 | 0.00 | 0.00 | 0.00 |
| PEDANIOS | 7 | 0.11 | 0.29 | 0.10 | 0.18 | 0.20 | 0.36 | 0.67 | 0.33 | 0.53 | 0.42 | 1.12 | 0.65 | 0.00 | 0.00 |

**Table S3.** Phytocannabinoids concentrations (% w/w, mean and SD) in Cannabis sativa oil preparations obtained using Method C [28-29] for the extraction of analytes from plant materials. In the first column are presented the different Cannabis varieties or some combinations among them.

| **SIFAP (n. 2323)** | **n.** | **CBDA** | **±SD** | **CBD** | **±SD** | **CBD tot** | **±SD** | **THCA** | **±SD** | **THC** | **±SD** | **THC tot** | **±SD** | **CBN** | **±SD** |
| --- | --- | --- | --- | --- | --- | --- | --- | --- | --- | --- | --- | --- | --- | --- | --- |
| AURORA | 5 | 0.04 | 0.05 | 0.22 | 0.32 | 0.25 | 0.37 | 0.17 | 0.16 | 0.80 | 0.73 | 0.95 | 0.87 | 0.00 | 0.00 |
| BEDICA | 39 | 0.00 | 0.00 | 0.10 | 0.34 | 0.10 | 0.34 | 0.09 | 0.16 | 0.98 | 0.27 | 1.06 | 0.25 | 0.00 | 0.01 |
| BEDIOL | 838 | 0.19 | 0.15 | 0.52 | 0.16 | 0.69 | 0.15 | 0.02 | 0.05 | 0.43 | 0.10 | 0.44 | 0.09 | 0.00 | 0.00 |
| BEDIOL+BEDROCAN | 10 | 0.01 | 0.02 | 0.31 | 0.02 | 0.33 | 0.02 | 0.00 | 0.00 | 0.89 | 0.06 | 0.89 | 0.06 | 0.00 | 0.00 |
| BEDIOL+BEDROLITE | 5 | 0.08 | 0.13 | 0.51 | 0.11 | 0.59 | 0.06 | 0.00 | 0.00 | 0.18 | 0.04 | 0.18 | 0.04 | 0.00 | 0.00 |
| BEDROBINOL | 1 | 0.00 | 0.00 | 0.07 | 0.00 | 0.07 | 0.00 | 0.03 | 0.00 | 0.76 | 0.00 | 0.78 | 0.00 | 0.00 | 0.00 |
| BEDROCAN | 800 | 0.00 | 0.03 | 0.01 | 0.11 | 0.01 | 0.12 | 0.19 | 0.29 | 1.33 | 0.36 | 1.49 | 0.34 | 0.00 | 0.01 |
| BEDROCAN+BEDIOL | 21 | 0.01 | 0.04 | 0.28 | 0.06 | 0.29 | 0.06 | 0.01 | 0.05 | 0.97 | 0.11 | 0.98 | 0.09 | 0.00 | 0.00 |
| BEDROCAN+BEDROLITE | 26 | 0.12 | 0.18 | 0.34 | 0.38 | 0.45 | 0.33 | 0.33 | 0.40 | 0.27 | 0.26 | 0.56 | 0.49 | 0.00 | 0.00 |
| BEDROCAN+FM2 | 1 | 0.00 | 0.00 | 0.13 | 0.00 | 0.13 | 0.00 | 0.00 | 0.00 | 1.06 | 0.00 | 1.06 | 0.00 | 0.00 | 0.00 |
| BEDROLITE | 151 | 0.15 | 0.11 | 0.50 | 0.18 | 0.63 | 0.19 | 0.00 | 0.00 | 0.01 | 0.05 | 0.01 | 0.05 | 0.00 | 0.01 |
| CBD | 1 | 0.00 | 0.00 | 2.92 | 0.00 | 2.92 | 0.00 | 0.00 | 0.00 | 0.00 | 0.00 | 0.00 | 0.00 | 0.00 | 0.00 |
| FM1 | 19 | 0.00 | 0.00 | 0.00 | 0.00 | 0.00 | 0.00 | 0.01 | 0.02 | 0.98 | 0.19 | 0.99 | 0.19 | 0.00 | 0.00 |
| FM2 | 352 | 0.10 | 0.13 | 0.79 | 0.20 | 0.88 | 0.18 | 0.01 | 0.03 | 0.55 | 0.11 | 0.56 | 0.11 | 0.00 | 0.01 |
| FM2+BEDROLITE | 1 | 0.00 | 0.00 | 0.80 | 0.00 | 0.80 | 0.00 | 0.00 | 0.00 | 0.36 | 0.00 | 0.36 | 0.00 | 0.00 | 0.00 |
| PEDANIOS | 37 | 0.02 | 0.09 | 0.07 | 0.20 | 0.08 | 0.25 | 0.18 | 0.15 | 1.33 | 0.51 | 1.49 | 0.57 | 0.00 | 0.01 |
| High THC | 11 | 0.00 | 0.01 | 0.00 | 0.00 | 0.00 | 0.01 | 0.14 | 0.29 | 1.68 | 0.49 | 1.80 | 0.49 | 0.00 | 0.01 |

**Table S4.** Phytocannabinoids concentrations (% w/w, mean ±SD) in Cannabis sativa oil preparations obtained using Method D [30] for the extraction of analytes from plant materials. In the first column are presented the different Cannabis varieties or some combinations among them.

| **CALVI (n. 428)** | **n.** | **CBDA** | **±SD** | **CBD** | **±SD** | **CBD tot** | **±SD** | **THCA** | **±SD** | **THC** | **±SD** | **THC tot** | **±SD** | **CBN** | **±SD** |
| --- | --- | --- | --- | --- | --- | --- | --- | --- | --- | --- | --- | --- | --- | --- | --- |
| BEDICA | 53 | 0.00 | 0.00 | 0.47 | 0.98 | 0.47 | 0.98 | 0.00 | 0.02 | 0.85 | 0.70 | 0.86 | 0.70 | 0.00 | 0.01 |
| BEDIOL | 86 | 0.05 | 0.14 | 0.63 | 0.49 | 0.67 | 0.49 | 0.01 | 0.06 | 0.34 | 0.10 | 0.35 | 0.11 | 0.00 | 0.00 |
| BEDROCAN | 151 | 0.00 | 0.00 | 0.24 | 0.56 | 0.07 | 0.54 | 0.01 | 0.04 | 1.24 | 0.51 | 1.24 | 0.52 | 0.01 | 0.03 |
| BEDROCAN + BEDROLITE | 1 | 0.07 | 0.00 | 0.21 | 0.00 | 0.27 | 0.00 | 0.00 | 0.00 | 0.01 | 0.00 | 0.01 | 0.00 | 0.00 | 0.00 |
| BEDROLITE | 53 | 0.11 | 0.24 | 0.58 | 0.53 | 0.68 | 0.50 | 0.00 | 0.00 | 0.01 | 0.06 | 0.01 | 0.06 | 0.00 | 0.00 |
| FM2 | 63 | 0.01 | 0.06 | 0.79 | 0.16 | 0.80 | 0.15 | 0.00 | 0.02 | 0.47 | 0.08 | 0.47 | 0.08 | 0.00 | 0.01 |
| PEDANIOS | 21 | 0.00 | 0.00 | 0.00 | 0.00 | 0.00 | 0.00 | 0.06 | 0.29 | 1.52 | 0.53 | 1.58 | 0.49 | 0.00 | 0.01 |

**Table S5.** Comparison between theoretical and experimental cannabinoids concentrations. The theoretical concentrations were considered as the mean of the declared range content and calculated as the 1:10 of the Cannabis varieties.

|  | **THC tot (% w/w)** | | **CBD tot (% w/w)** | |
| --- | --- | --- | --- | --- |
| **Cannabis products** | **Theoretical (mean, range)** | **Experimental** | **Theoretical (mean, range)** | **Experimental** |
| Bedrocan | 2.05, 1.9-2.2 | 1.47±0.47 | - | 0.04±0.31 |
| Bedrolite | - | 0.01±0.09 | 0.85, 0.7-1.0 | 0.66±0.35 |
| Bediol | 0.65, 0.5-0.8 | 0.45±0.26 | 0.75, 0.6-0.9 | 0.70±0.44 |
| FM-2 | 0.65, 0.5-0.8 | 0.54±0.12 | 1.05, 0.9-1.2 | 0.89±0.29 |

**Table S6.** Comparison between theoretical and experimental cannabinoids extraction efficiency as a function of preparation methods (EE%= conc. Experimental/ conc. Theoretical x100). The theoretical concentrations were considered as the mean of the declared range content and calculated as the 1:10 of the Cannabis varieties.

|  | **THC tot** | | **CBD tot** | |
| --- | --- | --- | --- | --- |
| **Cannabis products** | **Theoretical (% w/w; mean, range)** | **Experimental EE**% | **Theoretical (% w/w; mean, range)** | **Experimental EE**% |
| **Bedrocan** | **2.05, 1.9-2.2** |  | **-** |  |
| *Method A* |  | 74.48±20.62 |  | - |
| *Method B* |  | 72.72±21.70 |  | - |
| *Method C* |  | 72.88±16.56 |  | - |
| *Method D* |  | 60.64±25.30 |  | - |
| **Bedrolite** | **-** |  | **0.85, 0.7-1.0** |  |
| *Method A* |  | - |  | 73.43±28.95 |
| *Method B* |  | - |  | 57.06± 8.57 |
| *Method C* |  | - |  | 75.24±22.86 |
| *Method D* |  | - |  | 60.09±29.59 |
| **Bediol** | **0.65, 0.5-0.8** |  | **0.75, 0.6-0.9** |  |
| *Method A* |  | 67.71±24.24 |  | 85.49±31.35 |
| *Method B* |  | 68.81±14.94 |  | 90.59±22.53 |
| *Method C* |  | 68.86±14.56 |  | 92.76±21.52 |
| *Method D* |  | 57.68±21.62 |  | 79.50±26.68 |
| **FM-2** | **0.65, 0.5-0.8** |  | **1.05, 0.9-1.2** |  |
| *Method A* |  | 84.64±19.09 |  | 84.72±19.05 |
| *Method B* |  | 78.90±16.88 |  | 82.90±17.97 |
| *Method C* |  | 86.73±17.34 |  | 84.58±17.48 |
| *Method D* |  | 73.66±22.26 |  | 78.51±24.49 |

For details on preparation methods see the following references: Romano-Hazekamp (method A [26]), Cannazza (method B [27]), Sifap (method C [28,29]) and Calvi (method D [30]).

| 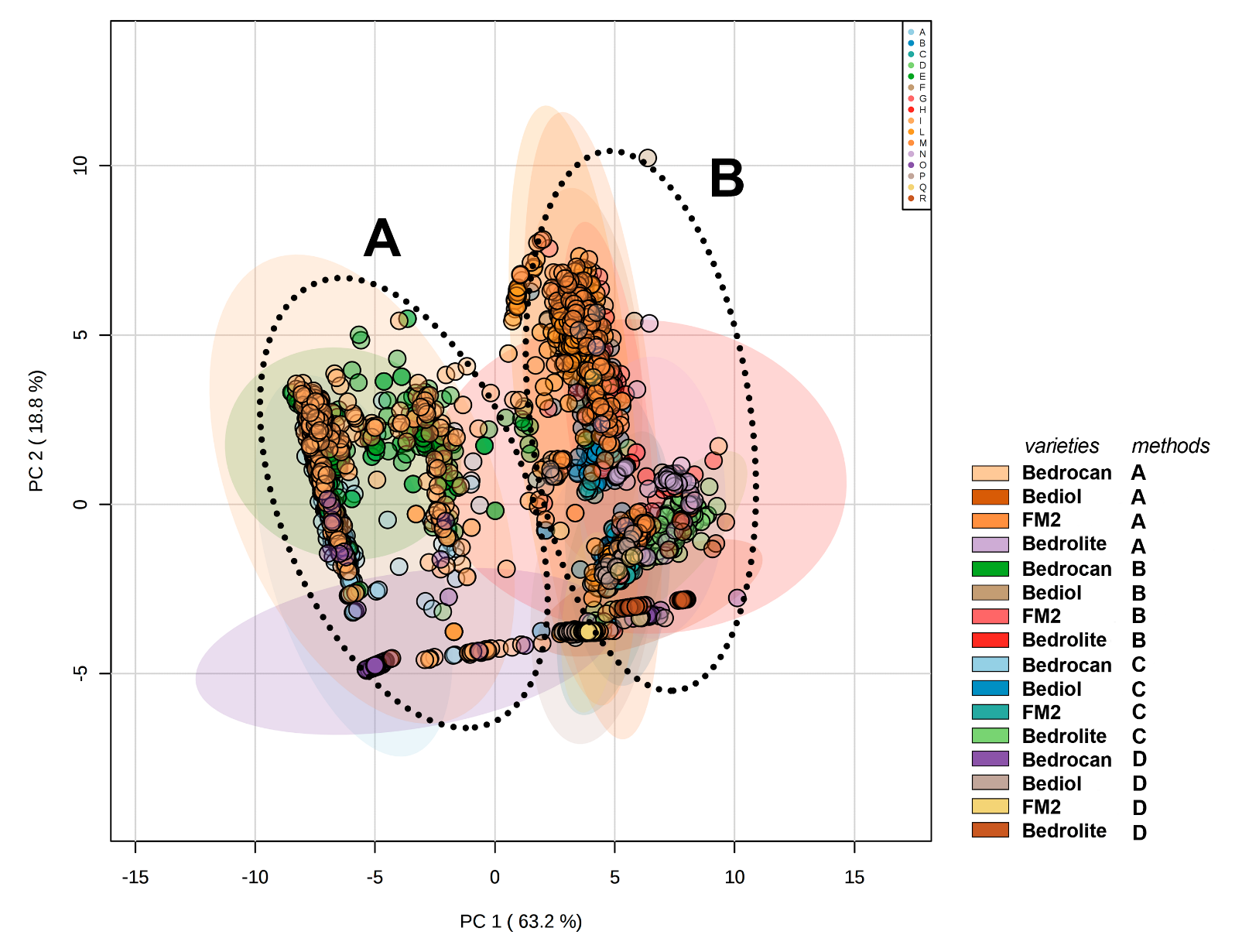 |
| --- |
| **Figure S1.** 2D PCA plot showing a separation of 63.2% on PC1 (n=4774). The ellipse colored-shaded areas indicate the 95% confidence regions based on the data points for individual groups. An appreciable separation can be distinguished by the two dotted areas: (**A**) Bedrocan and (**B**) other varieties: Bediol, Bedrolite and FM2. For details on preparation methods see the following references: Romano-Hazekamp (method A [26]), Cannazza (method B [27]), Sifap (method C [28,29]) and Calvi (method D [30]). |


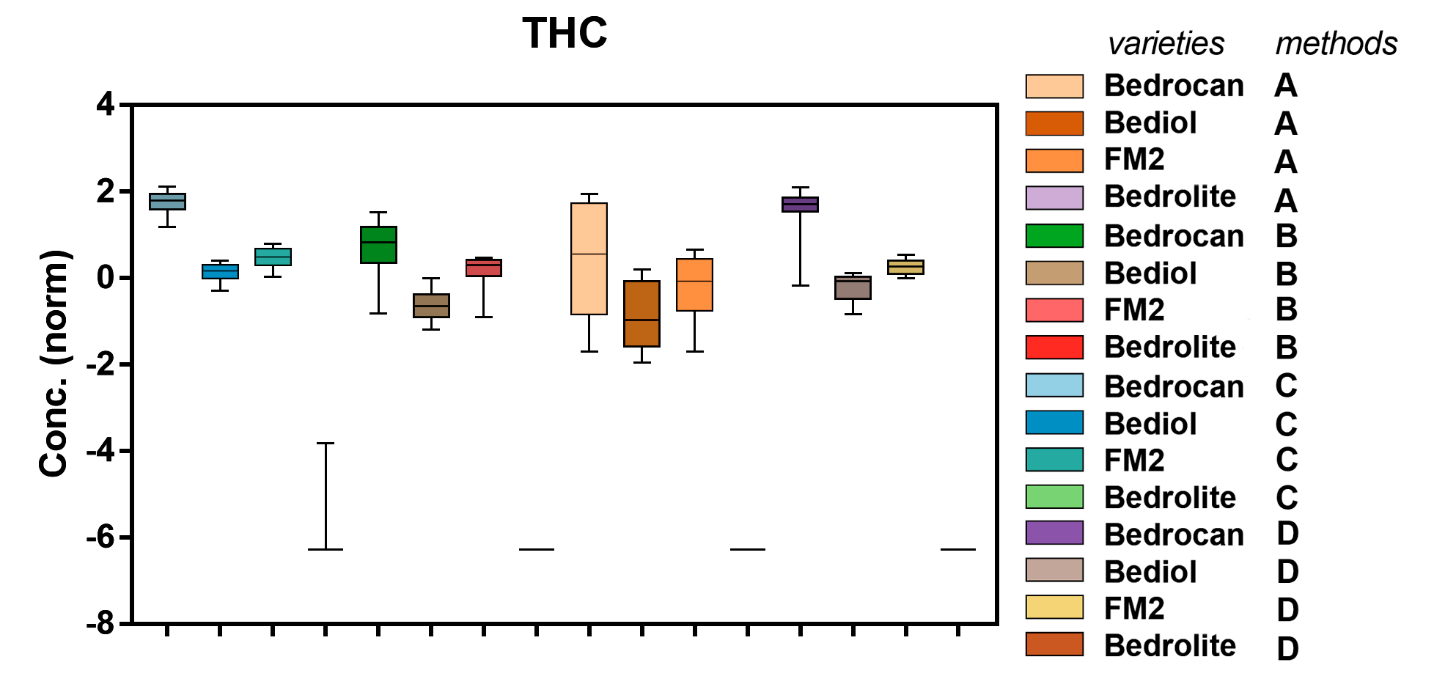


**Figure S2.** THC concentrations (after log-normalization and mean scaled) between different groups. Visualization by box and whiskers plot: the box extends from the 25th to 75th percentiles, the line in the middle is plotted at the median and whiskers are drawn down to the 10th percentile and up to the 90th. For details on preparation methods see the following references: Romano-Hazekamp (method A [26]), Cannazza (method B [27]), Sifap (method C [28,29]) and Calvi (method D [30]).
